# High anisotropy in electrical and thermal conductivity through the design of aerogel-like superlattice (NaOH)_0.5_NbSe_2_

**DOI:** 10.1038/s41467-023-42510-0

**Published:** 2023-10-21

**Authors:** Ruijin Sun, Jun Deng, Xiaowei Wu, Munan Hao, Ke Ma, Yuxin Ma, Changchun Zhao, Dezhong Meng, Xiaoyu Ji, Yiyang Ding, Yu Pang, Xin Qian, Ronggui Yang, Guodong Li, Zhilin Li, Linjie Dai, Tianping Ying, Huaizhou zhao, Shixuan Du, Gang Li, Shifeng Jin, Xiaolong Chen

**Affiliations:** 1 School of Science, China University of Geosciences, Beijing (CUGB), 100083 Beijing, China; 2 Institute of Physics, Chinese Academy of Science, 100190 Beijing, China; 3 School of Physical Sciences, University of Chinese Academy of Sciences, 100190 Beijing, China; 4 School of Physics, Liaoning University, 110136 Shenyang, China; 5 Department of Physics, Imperial College London, London, SW7 2AZ UK; 6 School of Energy and Power Engineering, Huazhong University of Science and Technology, 430074 Wuhan, China; 7 Cavendish Laboratory, 19 JJ Thomson Avenue, Cambridge, CB3 0HE UK; 8 Songshan Lake Materials Laboratory, 523808 Dongguan, China

**Keywords:** Superconducting properties and materials, Phase transitions and critical phenomena

## Abstract

Interlayer decoupling plays an essential role in realizing unprecedented properties in atomically thin materials, but it remains relatively unexplored in the bulk. It is unclear how to realize a large crystal that behaves as its monolayer counterpart by artificial manipulation. Here, we construct a superlattice consisting of alternating layers of NbSe_2_ and highly porous hydroxide, as a proof of principle for realizing interlayer decoupling in bulk materials. In (NaOH)_0.5_NbSe_2_, the electric decoupling is manifested by an ideal 1D insulating state along the interlayer direction. Vibration decoupling is demonstrated through the absence of interlayer models in the Raman spectrum, dominant local modes in heat capacity, low interlayer coupling energy and out-of-plane thermal conductivity (0.28 W/mK at RT) that are reduced to a few percent of NbSe_2_’s. Consequently, a drastic enhancement of CDW transition temperature (>110 K) and Pauling-breaking 2D superconductivity is observed, suggesting that the bulk crystal behaves similarly to an exfoliated NbSe_2_ monolayer. Our findings provide a route to achieve intrinsic 2D properties on a large-scale without exfoliation.

## Abstract (web summary)

Interlayer decoupling plays an essential role in realizing unprecedented properties. Here, authors construct a superlattice consisting of alternating layers of NbSe2 and highly porous hydroxide, realizing interlayer decoupling and thus realizing exotic monolayer behaviors in bulk materials.

## Introduction

Since the discovery of graphene^1^, atomic-thick materials have attracted tremendous interest in the past decades^2–5^. Compared to the bulk, isolated monolayers show a rich variety of distinct properties owing to their interlayer decoupling, both electrically and vibrationally. For instance, graphene displays exceptional electronic transport properties and stiffness^6^. Monolayer NbSe_2_ host exotic electronic states, including drastically enhanced charge-density wave (CDW) order and Ising superconductivity^7,8^. Black phosphorus^9^ and MoS_2_^2^ undergo indirect to direct band gap transitions as their thickness is reduced to the monolayer limit. These examples demonstrate that interlayer decoupling significantly impacts mechanical, electrical, and optical properties. However, when considering aspects such as fabrication, chemical and environmental stability, and sample quantity, bulk materials continue to outperform their monolayer counterparts. Therefore, it is highly desired to find a route to realize interlayer decoupling in large crystals, so that intrinsic 2D properties can be realized in bulk materials.

**Fig. 1:**
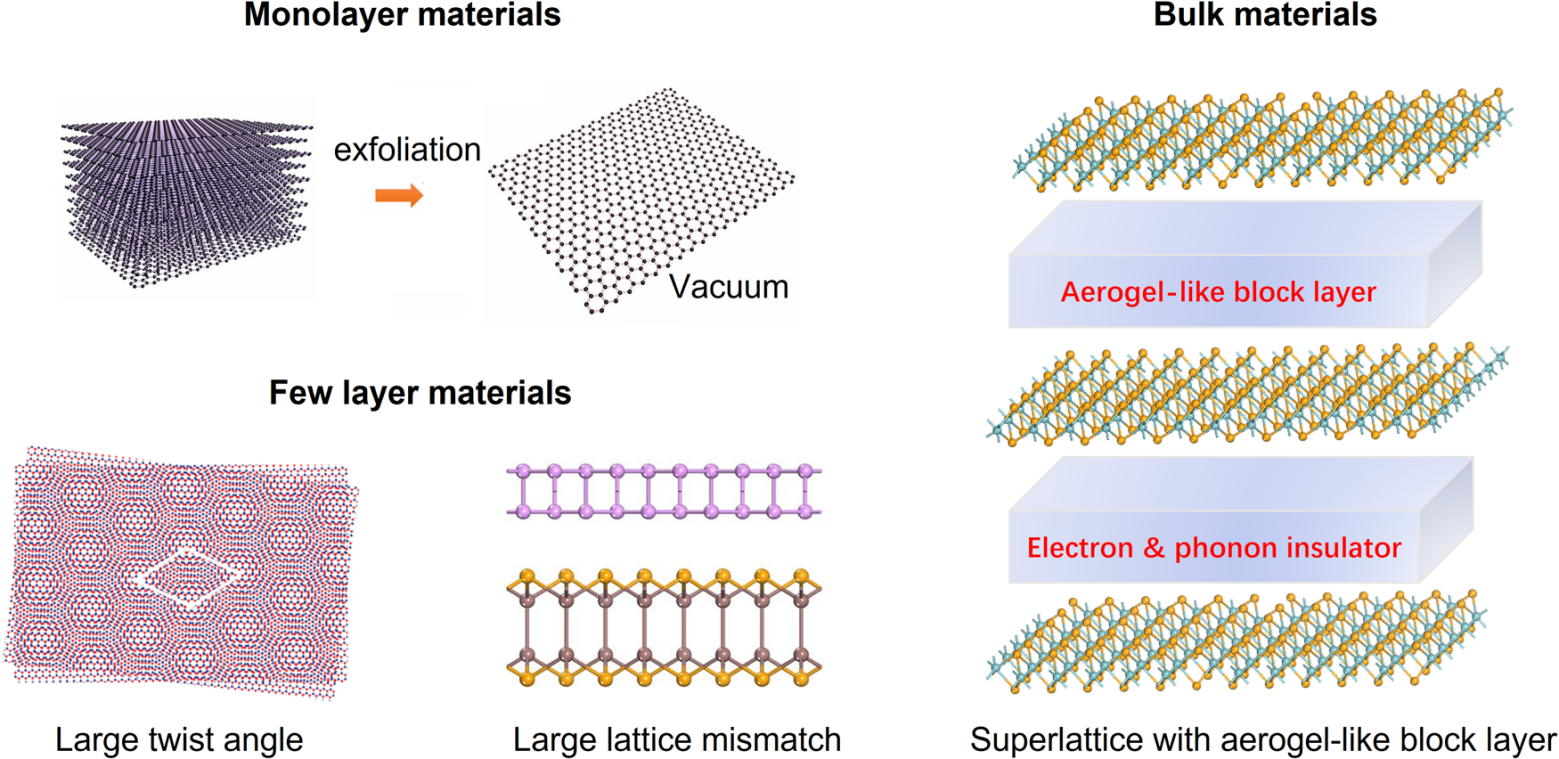
Interlayer decoupling schemes for different materials. The design scheme for achieving interlayer decoupling through exfoliating monolayers, introducing large twist angle and lattice mismatch in a few atomic layers, and incorporating aerogel-like block layers in bulk materials.

At present, feasible routes have been developed to realize interlayer decoupling in monolayer or few-layer 2D materials^10–14^. However, this ability is notably absent in bulk materials. For an exfoliated monolayer, the vibrational and electrical connections to the bulk are interrupted by the surrounding vacuum or air (Fig. 1a). In the case of stacked bilayer or thicker few atomic-layer materials, the interlayer coupling can be partially removed by increasing the twist angle^15,16^ or introducing lattice mismatch^17^ (Fig. 1b). However, in conventional bulk crystals, their periodic symmetry does not accommodate variations in either twist angle or lattice mismatch. Although artificially decoupling a bulk crystal remains a challenge, computational chemistry regularly obtains properties of decoupled 2D monolayers by introducing thick (~2 nm) vacuum layers into the periodic lattice of bulk materials. This vacuum layer, which inhibits the interlayer transmission of both electrons and phonons, serves as an ideal block layer for decoupling the bulk crystal into isolated monolayers. In practical applications, if the vacuum layer could be replaced by materials that are equally insulating to electrons and phonons, perfect decoupling of the bulk material should be achievable. Recently, this concept was partially demonstrated in a bulk superlattice, Ba_6_Nb_11_S_28_, wherein the metallic NbS_2_ layers were spatially separated by 1 nm thick Ba_3_NbS_5_ block layers, resulting in clean 2D superconductivity^18^. However, akin to other metals inserted with insulating block layers, metallic behavior persists along the interlayer direction of Ba_6_Nb_11_S_28_, indicating imperfect electron decoupling. To the best of our knowledge, there is currently no evidence that traditional block layers can prevent phonon transmissions and decouple the bulk material vibrationally.

In practice, few solid materials share the same capability of blocking phonons as the vacuum. For example, it is well known that the thermal conductance of a crystalline material, which is mediated by non-localized phonons, cannot be arbitrarily low^19^. Surprisingly, for some highly-porous-and-low-density materials, such as aerogels, their thermal conductivity (12 mW m^−1^ K^−1^) can be even lower than that of air^20–22^. Inspired by the extraordinarily hollow and disordered structure of aerogels, we construct some hydroxide layers with extremely high porosity, i.e., up to 95% of the volume in some hydroxide layers remains vacant. These fluffy aerogel-like layers (~2 nm) facilitate the lattice matching with a variety of transition metal dichalcogenides (TMDs) and give rise to a new family of bulk superlattices (Fig. S1). In particular, (NaOH)_0.5_NbSe_2_, one of the superlattices with alternating porous NaOH block layers and NbSe_2_ monolayers, is selected as a model system to investigate the effect of artificial interlayer decoupling in bulk materials (Fig. 1c).

NbSe_2_ is one of the most studied van der Waals materials, and meanwhile, a model system for investigating the effect of dimensionality on correlated electron-phonon phenomena. In bulk NbSe_2_, a CDW sets in at ${T}_{{CDW}}=33\,{{{{{\rm{K}}}}}}$, and superconductivity sets in at a critical temperature ${T}_{C}=7.2{{{{{\rm{K}}}}}}$^23^. In the monolayer limit, however, a highly unusual enhancement of T_CDW_ is observed, and a Pauli-breaking Ising superconductivity set in at 1.9 K^7,8,24^. In this work, we show that those exotic monolayer behaviors are indeed realized in large single crystals of (NaOH)_0.5_NbSe_2_. Systematic investigations suggest that the thick and porous NaOH layers act as superinsulators and detach the NbSe_2_ monolayers spatially, electrically and vibrationally, as confirmed by X-ray diffraction, Raman spectrum, heat capacity, heat transport measurements, orientation and temperature-dependent resistivity, and density functional theory (DFT) calculations. Moreover, the fitting of angle-dependent magnetic resistance and magnetic torque data reveal that the exotic superconductivity and CDW states in bulk (NaOH)_0.5_NbSe_2_ are consistent with those found in NbSe_2_ monolayer, suggesting this decoupled bulk crystal act just like an exfoliated NbSe_2_ monolayer. The concept of introducing aerogel-like layers to decouple the bulk materials can be easily extended to other TMDs materials (Fig. S1) and even beyond (graphite, Xenes, BN, etc.), which may bring us to a new era of unconventional bulk materials with unprecedented electrical, mechanical and optical properties.

## Results and discussion

### Chemical compositions and crystal structure

**Fig. 2:**
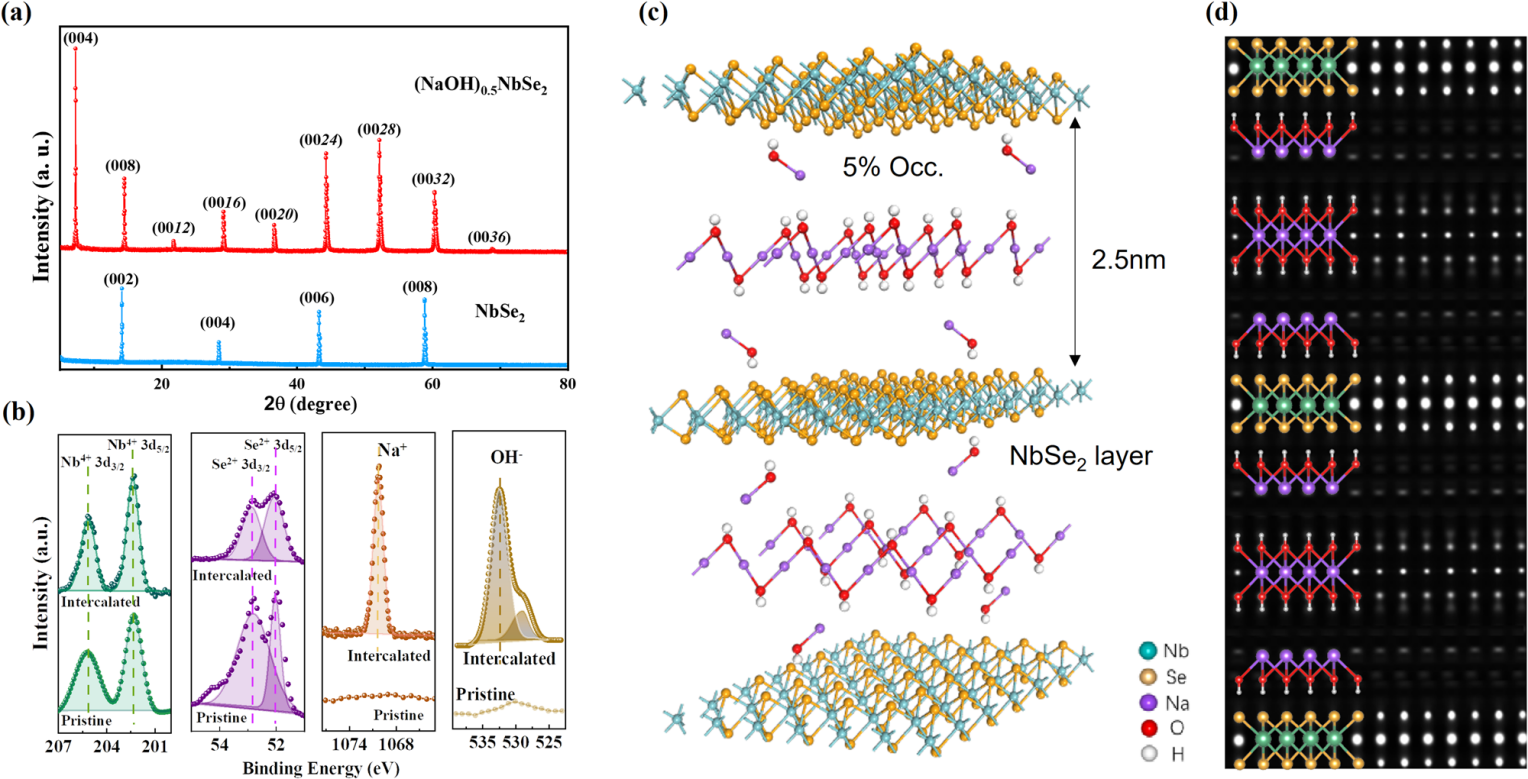
Structure characterization of (NaOH)_0.5_NbSe_2_. **a** Powder X-ray diffraction patterns collected for NbSe_2_ and (NaOH)_0.5_NbSe_2_ single crystals. **b** XPS pattern of Nb, Se, and Na, OH^-^ for (NaOH)_0.5_NbSe_2_. **c** Crystal structure of (NaOH)_0.5_NbSe_2_. **d** Experimental charge density of (NaOH)_0.5_NbSe.

As shown in Figs. 2a and S1, single crystals of (AOH)_x_MX_2_ (A = Na, K; M = Ta, Nb; X = S, Se) up to 0.6 cm in size were synthesized under hydrothermal conditions by using MX_2_ single crystals as the precursor (detailed in methods). In the case of (NaOH)_0.5_NbSe_2_, the X-ray diffraction data in Fig. 2a show that the (00l) reflections of the NbSe_2_ precursors are completely replaced by a new set of ones that systematically shift to lower angles, suggesting the incorporation of new block layers into the NbSe_2_ layered structure. Figure S2 shows the scanning electron microscopy (SEM) image for the as-prepared crystal, EDS mapping reveals a homogeneous distribution of Na, Nb, and Se atoms after the reaction. The atomic ratio of Na: Nb: Se is determined to be 0.5:1:2, so the concentration x of Na-containing species should be half of the NbSe_2_. XPS analysis was carried out on a freshly cleaved single crystal to probe the species and the oxidation states of the elements in NbSe_2_ and (NaOH)_0.5_NbSe_2_, with the corresponding Nb*3d*, Se*3d*, Na*1s*, and OH^-^ spectra presented in Fig. 2b. The Nb*3d* and Se*3d* spectra of pristine and the intercalated NbSe_2_ have identical binding energies, indicating the oxidization state of NbSe_2_ is unaltered upon reaction, and there is no evidence of charge transfer between the block layer and the NbSe_2_ matrix. Meanwhile, a sharp peak at 1070 eV was ascribed to the Na^+^^25^, and the peak at 532.4 eV was assigned to OH^-^, confirming the NaOH molecules as the intercalants. The shoulder peak at 529.1 eV corresponded to a trace amount of adsorbed water molecules, rather than hydrated water in the structure^26^. From this, the chemical formula of the intercalated samples can be best described as (NaOH)_0.5_NbSe_2_.

To determine the crystal structures of (NaOH)_0.5_NbSe_2_, single-crystal X-ray diffraction (SXRD) analysis was implemented on a small specimen (132 × 74 × 17 μm^3^). The SXRD diffraction data can be indexed by a trigonal unit cell (space group P-3m1), with lattice parameters *a* = 3.4554 Å, *c* = 48.9648 Å (Fig. 2c). Form the high accuracy experimental electron density constructed via. the model independent Maximum entropy method (Fig. 2d), the NaOH molecules can be located at the 1a (0, 0, 0), 1b (0, 0, 1/2), 2c (0, 0, z), and 2d (2/3, 1/3, *z*) sites, which form ~2 nm thick NaOH trilayers in between the NbSe_2_ monolayers. It is worth noting that the resolved electron density of the two outmost NaOH layers is much lower (Fig. 2d), suggesting all the sites neighboring the NbSe_2_ layers have much smaller occupancy factors. Based on the resolved structure model, the crystal structure refinements converged rapidly to small residuals $R1=0.0313$. The final crystal structures and the refined structural parameters are shown in Fig. 2c & d and Table S1, and the bond lengths and angles in (NaOH)_0.5_NbSe_2_ are close to that of 2H-NbSe_2_ and NaOH (Table S2). As shown in Fig. 2c & d, the extraordinarily large NbSe_2_ interlayer distance ($d=24.5{{{{{\text{\AA}}}}}}$) is up to four times that of pristine NbSe_2_ (6.14 Å), and two times that of most known intercalated NbX_2_ (X = S, Se) compounds^27^, confirming the NbSe_2_ monolayers are well spatially detached. More strikingly, structure refinement suggests only 5 % of the sites in the two outmost layers are randomly occupied by NaOH molecules, consistent with the results of charge density analysis (Fig. 2c, d). The density of the extremely low occupied two NaOH layers surrounding the NbSe_2_ layer is merely 0.08 g cm^−3^, comfortably staying in the typical density range of aerogels (C.A. 0.0011 to ~0.5 g cm^−3^). The occupancy of the central NaOH layer reaches 40 %, which is necessary to brace up the structure framework.

### The uniaxial-insulating behavior and electrical decoupling

**Fig. 3:**
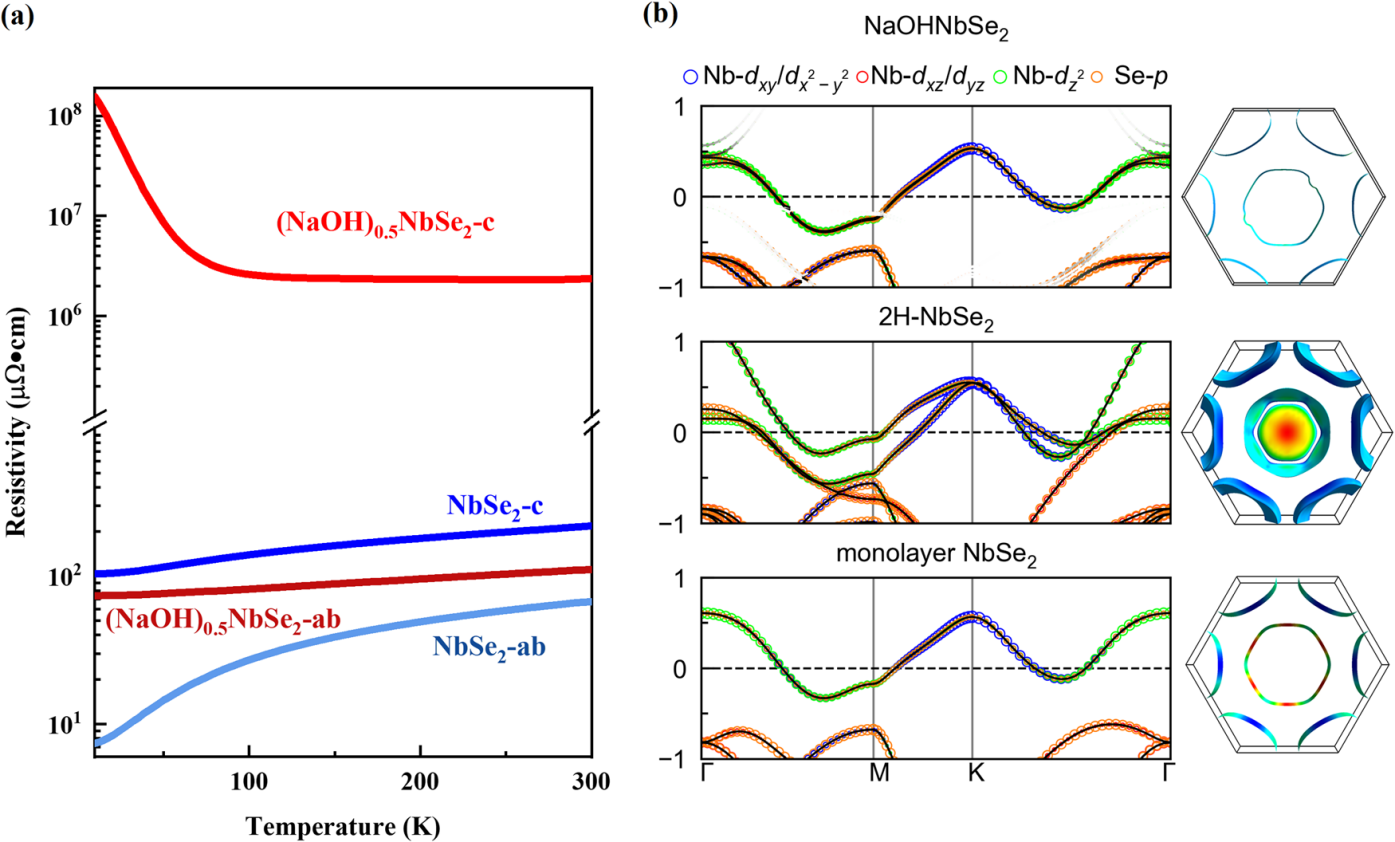
The uniaxial-insulating behavior and band structure of (NaOH)_0.5_NbSe_2_. **a** The temperature-dependent in-plane and out-plane resistivity measured for NbSe_2_ and (NaOH)_0.5_NbSe_2_ between 3 and 300 K. **b** The band structures and Fermi surfaces of (NaOH)_0.5_NbSe_2_, bulk, and monolayer NbSe_2_. The size of the circles is proportional to the contribution of NbSe_2_.

Electrical anisotropy is generally small in metallic crystals. However, in the case of a fully decoupled metal, an insulating behavior is expected along the interlayer direction, whereas metallic behavior should be retained for in-plane directions. The MX_2_(M = Ta, Nb, X = S, Se) single crystals used here are all metallic^28^, and the reported electrical anisotropy $\beta={\rho }_{{ab}}\left(T\right)/{\rho }_{c}\left(T\right)$ is lower than 200 (*ρ*_*ab*_*(T)* and *ρ*_*c*_*(T)* are in-plane and out-plane resistivities, respectively)^29^. By inserting ~ 2 nm thick AOH layers in between the MX_2_ layers, giant electronic anisotropy appeared in all (AOH)_x_MX_2_ materials, which behave as either band insulators or metals, depending on the measured crystal axis (Figs. S3 and S4a). Figures 3a, S4b show the *ρ*_*ab*_*(T)* and *ρ*_*c*_*(T)* data of NbSe_2_ and (NaOH)_0.5_NbSe_2_ measured between 3 K and 300 K. Along both a- and c- directions, the NbSe_2_ single crystal show metallic behavior, where the measured β was negligibly small - less than 2.5 at 300 K. At 7 K, a superconducting transition is observed (Fig. S4b). After incorporating the thick NaOH block layers, the (NaOH)_0.5_NbSe_2_ crystal remains metallic along the a-direction, and the resistivity increases slightly compared to that of NbSe_2_. Along the c-axis, however, the resistivity increases drastically—up to 10^6^ times that of NbSe_2_. Furthermore, the rapid enhancement of *ρ*_*c*_(*T*) at low temperature can be well fitted by a semiconducting thermal activation model (Fig. S3). Interestingly, this exotic axial-dependent insulating behavior is observed in all the measured (AOH)_x_MX_2_ materials, as shown in Fig. S3. In Fig. S5, the resistance anisotropy *β* of various TMDs materials are presented. The *β* of the metallic TMDs (NbSe_2_, TaS_2_, etc.) is very small, generally in the order of ~10, while their *β* can be further increased to ~10^3^−10^4^ by the intercalation of molecules or inorganic layers^30,31^. The recent incorporation of 1 nm thick, stoichiometric Ba_3_NbS_5_ layers in quasi-2D superlattice Ba_6_Nb_11_S_28_^18^ increased the *β* values to around 10^3^, which is three orders of magnitudes smaller than the maximum value realized in (NaOH)_0.5_NbSe_2_. The huge resistance anisotropy and especially the insulating behavior of *ρ*_*c*_(*T*) suggest that the thick hydrate layers have effectively eliminated the interlayer electronic couplings between NbSe_2_ monolayers.

Figure 3b shows the DFT calculated band structures of (NaOH)_0.5_NbSe_2_, bulk NbSe_2_ and monolayer NbSe_2_. For (NaOH)_0.5_NbSe_2_, all bands crossing the Fermi level are contributed by the spatially detached NbSe_2_ layers, resulting in nearly identical band structures and fermi surfaces of (NaOH)_0.5_NbSe_2_ and monolayer NbSe_2_. In bulk NbSe_2_, the degeneracy of the Nb *3d* band that crosses the Fermi level is broken by the interlayer coupling with bonding/antibonding configurations of Se *p*_*z*_ orbitals, which gives rise to two split bands in the Γ-M-K-Γ plane and two concentric cylindrical Fermi surface. In the monolayer form, the interlayer coupling is absent, both bands shifting of Se *p*_*z*_ orbitals across the Fermi level and the band splitting of Nb *4d* orbitals are avoided, leaving only one Nb band across the Fermi level. In (NaOH)_0.5_NbSe_2_, the Se *p*_*z*_ conducting bands are found to sink below the Fermi energy, and the band degeneracy of Nb *4d* states is almost perfectly preserved, leading to a monolayer-like band structure without interlayer couplings^32^. Moreover, in bulk NbSe_2_, the antibonding between interlayer Se *p*_*z*_ orbitals lift the Se *3p* band across the Fermi level, creating a small Fermi surface with strong *k*_*z*_ dispersion. In (NaOH)_0.5_NbSe_2_, the band structure exhibits only the dispersionless flat bands along the Γ–A direction. Therefore, like monolayer NbSe_2_, the fermi surfaces of (NaOH)_0.5_NbSe_2_ are derived from a single (or degenerate) Nb *4d* band that is dispersionless along the *k*_*z*_ direction, confirming the elimination of interlayer electronic couplings.

### The vibrational and phonon decoupling

**Fig. 4:**
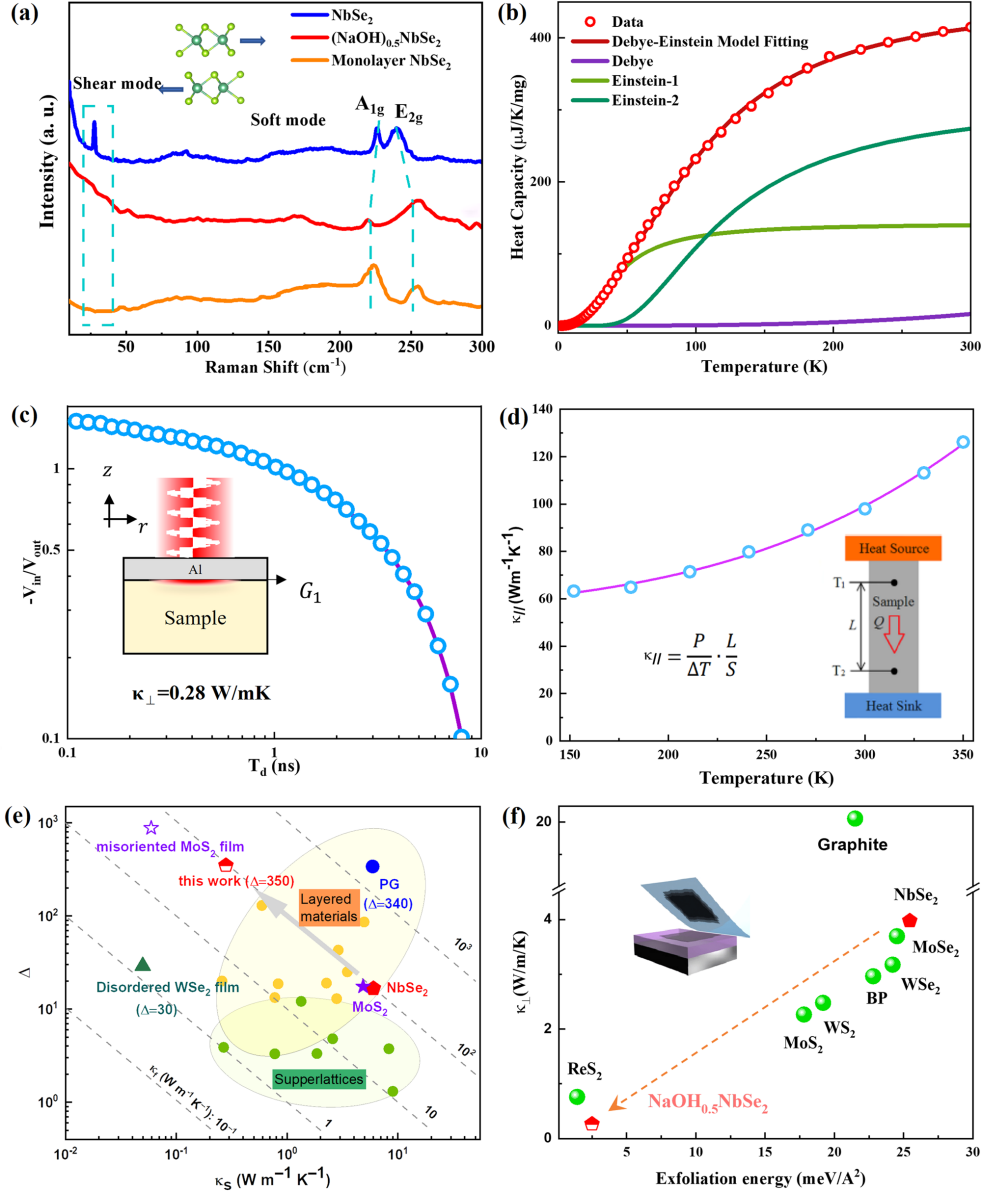
The vibrational and phonon decoupling behaviors of (NaOH)_0.5_NbSe_2_. **a** Room-temperature Raman spectra for bulk, monolayered NbSe_2_ and (NaOH)_0.5_NbSe_2_. **b** *C*_*p*_ vs. temperature plot in 2–300 K range. The solid blue line is calculated using combined Debye–Einstein model. The individual contributions from Debye (*β*) and the two Einstein terms are also plotted. **c** TDTR data for the out-of-plane thermal conductivity *κ*_*⊥*_ of (NaOH)_0.5_NbSe_2_. **d** the in-plane thermal conductivity *κ*_*//*_ of (NaOH)_0.5_NbSe_2_ measured by steady-state heat flow method. **e** Comparison of $\Delta={\kappa }_{//}/{\kappa }_{\perp }$, *κ*_//_ (x axis), and *κ*_*⊥*_ (diagonal dashed lines) measured for highly anisotropic thermal conductors and (NaOH)_0.5_NbSe_2_. Data of layered materials and superlattice are taken from Ref. ^38^. **f** *κ*_⊥_ vs. exfoliation energy diagram for typical TMDs materials, graphite, black phosphorus (BP) and (NaOH)_0.5_NbSe_2_^33,38–40,49–51^.

Besides electronic decoupling, we demonstrate that the numerous NbSe_2_ layers in (NaOH)_0.5_NbSe_2_ separated by sparse NaOH layers are also decoupled vibrationally. Figure 4a shows the room-temperature Raman spectra for bulk NbSe_2_, monolayer NbSe_2_^7^ and (NaOH)_0.5_NbSe_2_, respectively. The NbSe_2_ intra-layer vibration models, including E_2g_ mode at ~250 cm^−1^, A_1g_ mode at ~220 cm^−1^, and the soft mode at the range from 150 to 200 cm^−1^, were observed in all three samples. However, the most known interlayer vibration model in bulk and few-layer NbSe_2_, which manifest itself as a strong low-frequency vibrational band (20–30 cm^–1^), is absent in monolayer NbSe_2_ and (NaOH)_0.5_NbSe_2_. The absence of low-frequency shearing modes in the Raman spectrum of NbSe_2_ is only found in its monolayer form, implying that in (NaOH)_0.5_NbSe_2_, interlayer vibration couplings between NbSe_2_ monolayers are effectively eliminated by introducing thick aerogel-like NaOH layers.

The vibrationally decoupled substructures in (NaOH)_0.5_NbSe_2_ can be treated as independent harmonic oscillators (or Einstein oscillators), similar to clathrates and filled-skutterudites, whereas the remaining lattice can be treated within the Debye model. Figure 4b shows the temperature-dependent heat capacity *C*_*p*_ of (NaOH)_0.5_NbSe_2_ in the range of 2–300 K, with a fitting curve using a combined Debye–Einstein mode (detailed in supplementary materials). A minimum of two Einstein modes are required to adequately model the temperature dependence of *C*_*p*_, whose characteristic temperatures were determined to be ${\theta }_{E1}=128{K}$ and ${\theta }_{E2}=174{K}$ through fitting, with the estimated Debye temperature ${\theta }_{{Debye}}=237{K}$ (see the fitting parameters for Debye–Einstein model in Table S3). As shown in Fig. 4b, the harmonic Einstein oscillators contribute to 91% of the total specific heat, suggesting a substantial part of phonon vibrations in (NaOH)_0.5_NbSe_2_ is localized. Considering the chemical bonds within the NbSe_2_ layers are intact and strong, we believe the localized phonon is confined by the weak interlayer bonding between the sparse NaOH layers and NbSe_2_ layers, with the absence of up to 95% of the chemical bonds.

To experimentally verify the vibrational decoupling in (NaOH)_0.5_NbSe_2_ along the interlayer direction, the thermal conductivity of a (NaOH)_0.5_NbSe_2_ single crystal is measured using the beam-offset time domain thermos-reflectance (TDTR) method^33^. In Fig. 4c, we compare the ratio of the in-phase voltage (*V*_in_) to the out-of-phase voltage (*V*_out_) of the measured TDTR signals with the ratio calculated from a thermal model. As shown in Fig. 4c, at room temperature the out-of-plane thermal conductivity κ_⊥_ is determined to be merely 0.28 W m^−1^ K^−1^ within experimental uncertainties, which is drastically reduced to 7% of bulk NbSe_2_ (Fig. S6). The through-plane conductivity of (NaOH)_0.5_NbSe_2_ is among the lowest values achieved in bulk inorganic materials^34–37^. The sharp decline of *κ*_⊥_ in (NaOH)_0.5_NbSe_2_ can be attributed to the ultralow interlayer coupling strength of (NaOH)_0.5_NbSe_2_, which leads to the much-reduced group velocity of lattice vibrations along the out-of-plane direction. In order to evaluate the anisotropy of thermal conductivity, we measured the in-plane thermal conductivity *κ*_*∥*_ of (NaOH)_0.5_NbSe_2_ single crystal using a steady-state heat flow setup (Fig. 4d). The *κ*_∥_ of (NaOH)_0.5_NbSe_2_ was measured in the temperature range of 150–350 K, and the results are presented in Fig. 4d. At 300 K, the measured *κ*_∥_ is 98.1 W/mK, suggesting the thermal conductivity anisotropy ($\Delta={\kappa }_{//}/{\kappa }_{\perp }$) of (NaOH)_0.5_NbSe_2_ reaches an exceptionally high value of ~350. As shown in Fig. 4e, this large anisotropic value is second only to MoS_2_ thin films stacked by misoriented monolayers (Δ ≈ 880)^38^, and exceeds that of pyrolytic graphite (PG)—one of the most anisotropic bulk thermal conductors (Δ ≈ 340)^39^.

To get an insight into the observed interlayer decoupling at the atomic level, we have calculated the potential energy curves by elongating the interlayer distance between NbSe_2_ atomic layers, which gives the theoretical interlayer coupling energy E. As shown in Fig. S7a, the DFT calculations show that NbSe_2_ monolayers are indeed weakly coupled to NaOH layers in (NaOH)_0.5_NbSe_2_. The interlayer coupling energy E only reached 2.5 meV/ Å^2^, around 9 % of that of bulk NbSe_2_ (25 meV/Å^2^). Such a small E is even comparable to the low coupling energy that found in exfoliated monolayers deposited on different substrates^40^. Naturally, the low occupancy factor in aerogel-like NaOH layers (Occ = 0.05) is expected to reduce the strength of the vdWs interactions. To clarify how partial occupancy affects the interlayer coupling energy, we performed DFT calculations based on structure models with a range of Occ between 0.05 and 1.0. As shown in Fig. S7b, the interlayer energy E increased proportionally to Occ, and reached 29 meV/Å^2^ at Occ = 1.0, a value similar to that of pristine NbSe_2_. The findings suggest the ultralow density (or Occ) in aerogel-like NaOH layers plays a crucial role in reducing the interlayer coupling in (NaOH)_0.5_NbSe_2_. Figure 4f presents a survey of crystallized vdWs materials sorted according to their interlayer coupling energy and κ_⊥_, both of which should approach 0 when the material is perfectly decoupled. It shows that (NaOH)_0.5_NbSe_2_ exhibits both very low interlayer coupling energy and κ_⊥_, and more than 90% of them in pristine NbSe_2_ is removed by incorporating the highly porous NaOH layers, pushing (NaOH)_0.5_NbSe_2_ toward the best bulk material in terms of interlayer decoupling.

### The enhanced CDW and suppressed superconductivity

**Fig. 5:**
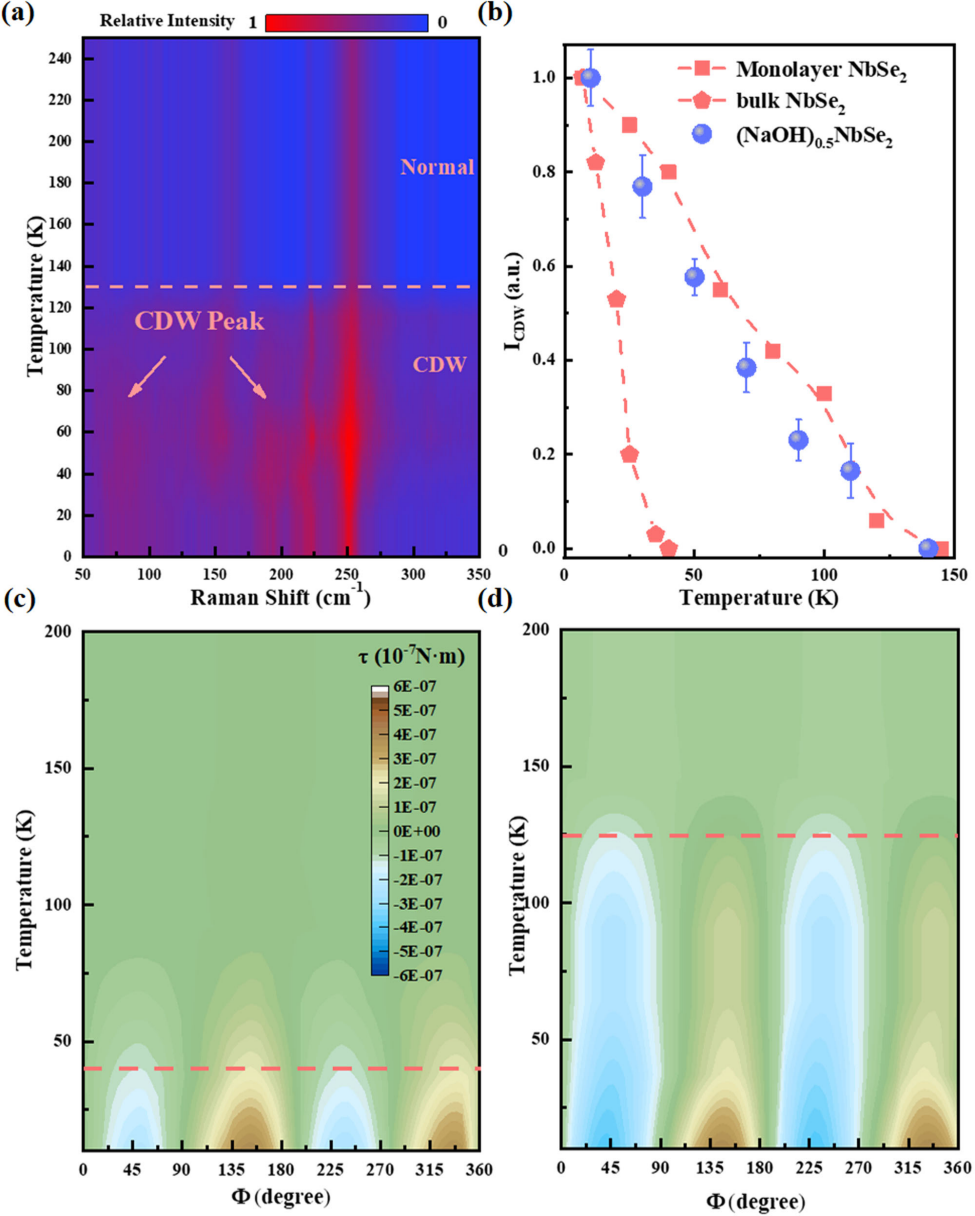
CDW characterization of (NaOH)_0.5_NbSe_2_ and NbSe_2_. **a** The temperature-dependent Raman spectra for (NaOH)_0.5_NbSe_2_. **b** Temperature dependence of the amplitude mode intensity *I*_CDW_ for (NaOH)_0.5_NbSe_2_ and reported monolayer NbSe_2_ results. **c**, **d** The angular-dependent magnetic torque under various temperatures for bulk (**c**) NbSe_2_ and (**d**) (NaOH)_0.5_NbSe_2_. The color mapping in the figure represents the torque value.

The CDW transition usually involves lattice distortion in which the electron-phonon coupling plays an important role. In bulk NbSe_2_, the CDW transition is generally below 33 K^7^. In monolayer NbSe_2_, the electron-phonon interactions are significantly enhanced in the 2D limit, leading to a remarkable enhancement of the charge density wave (CDW) phenomenon (${T}_{{CDW}}=145{{{{{\rm{K}}}}}}$)^7^. In line with this, Raman scattering experiments have revealed the emergence of two new phonon vibrational modes in the CDW state compared to the normal state. This is evident in the spectrum by the presence of two additional vibrational peaks (at 190 cm^−1^ and 75 cm^−1^) under T_CDW_. In Figs. 5a and S8, we show the temperature dependence of the Raman spectra measured for (NaOH)_0.5_NbSe_2_. From 300 K to 140 K, the spectra are relatively clean and agree well with monolayer NbSe_2_ above its *T*_*CDW*._ The prominent features below 300 cm^−^^1^ include an *E*_*2*_*g* mode around 250 cm^−1^, an A_1_g mode around 220 cm^−1^, and a soft model around 170 cm^−1^, with the absence of the interlayer shear model in bulk NbSe_2_. Below 140 K, two new modes appear in the Raman spectra. The first new mode emerged around 190 cm^−1^, and the second feature is a broad amplitude mode that appeared around 75 cm^−1^, both of which were observed in the CDW-state of bulk NbSe_2_ and monolayer NbSe_2_ below *T*_*CDW*_ and were attributed to the collective excitation of the CDW fluctuations. As shown in Fig. 5a, in (NaOH)_0.5_NbSe_2_, the CDW-related vibrational modes appeared much higher than 33 K, indicating an enhanced T_CDW_. Figure 5b shows the intensity *I*_*CDW*_ of the CDW-related model at 190 cm^−1^. The *T*_*CDW*_ of (NaOH)_0.5_NbSe_2_ should be below the temperature where *I*_*CDW*_ dropped to zero. The temperature dependence of *I*_*CDW*_ in bulk and monolayer NbSe_2_ taken from pieces of literature are also presented in Fig. 5b for comparison^7^. As for (NaOH)_0.5_NbSe_2_, the intensity of the CDW-related Raman peak vanishes between 110 and 140 K, and the significantly enhanced CDW order is in line with the findings in monolayer NbSe_2_. Moreover, the amplitude mode and folding mode corresponding to CDW in (NaOH)_0.5_NbSe_2_ are much stronger than in monolayer NbSe_2_^7^. Since the intensity of the Raman signal is proportional to the number of layers, the enhancement here is likely to be caused by the superposition of many monolayer signals.

With the advantages of bulk crystals, we can directly use torque magnetometry to reveal more details about the CDW in (NaOH)_0.5_NbSe_2_. Torque magnetometry measures the torque $\tau={\mu }_{0}V{{{{{\boldsymbol{M}}}}}}\times {{{{{\boldsymbol{H}}}}}}$ that a magnetic moment **M** experienced in a uniform magnetic field **H** (*μ*_*0*_ is the permeability of vacuum), where the $\tau$ value is proportional to sample volumes *V*. This technique is particularly useful for studying CDW systems because the CDW order often leads to changes in the magnetic susceptibility. In the case of H-NbSe_2_, the CDW order is known to arise within the Nb atomic layer. Both H-NbSe_2_ and (NaOH)_0.5_NbSe_2_ feature Nb atomic layers with a *D3h* symmetry, which can be equally described in an orthogonal lattice using new crystallographic axes, namely *a*_o_ and *b*_o_ (Fig. S9a). In the orthorhombic setting, the torque $\tau$ is simplified to a periodic function of the doubled azimuthal angle 2φ measured from the a-axis:

1 $${\tau }_{2\varphi }=\frac{1}{2}{\mu }_{0}{H}^{2}V\left[\left({\chi }_{{aa}}-{\chi }_{{bb}}\right)\sin 2\varphi -2{\chi }_{{ab}}\cos 2\varphi \right]$$

where the *χ*_*aa*_*, χ*_*bb*_ and *χ*_*ab*_ are magnetic susceptibility tensors in the orthorhombic setting. We performed torque measurements on 2H-NbSe_2_ and (NaOH)_0.5_NbSe_2_ single crystals using a setup depicted in Fig. S9b. The φ-scan torque values were recorded between 10 K and 200 K, and the corresponding angular-dependent torque curves are presented in Fig. S10. Notably, the angular-dependent torque curves of NbSe_2_ maintained a two-fold Sine function (with a period of π) throughout the CDW transition. This observation is consistent with the retention of *D3h* symmetry in the 3 × 3 CDW phase of NbSe_2_. Below the CDW transition temperature, we observed a significant increase in the amplitude of τ_2φ_, indicating enhanced magnetic anisotropy between different susceptibility tensors in the CDW phase (Fig. 5c). For (NaOH)_0.5_NbSe_2_, the φ-scan magnetic torque spectra in Fig. 5d show the same *sin(2φ)* form in both the normal state and below the *T*_*CDW*_ (as determined by Raman scattering), strongly suggesting that the CDW phase in (NaOH)_0.5_NbSe_2_ also retained the *D3h* symmetry as in the 3×3 CDW phase of H-NbSe_2_. Meanwhile, the drastic increase of magnetic torque strength, signifying the onset of CDW ordering, occurred at much higher temperatures in (NaOH)_0.5_NbSe_2_ (>120 K), in consistency with the Raman scattering results.

At last, we turn to the superconducting state of (NaOH)_0.5_NbSe_2_. Figure S11a shows a detailed view of the superconducting transition, which shows onset near $T=1.7{{{{{\rm{K}}}}}}$ and reaches zero resistance at $T=1.2{{{{{\rm{K}}}}}}$. The reduction in the transition temperature for the NbSe_2_ layers in (NaOH)_0.5_NbSe_2_ relative to bulk H-NbSe_2_ is consistent with NbSe_2_ monolayer (*T*_*c*_ = 1.5 K)^24^. Noticeably, the *ρ*_*ab*_*(T)* for (NaOH)_0.5_NbSe_2_ for 1.5 K < *T* < 1.8 K is well described by the Halperin-Nelson model^41^: ${\rho }_{{ab}}^{F}\left(T\right)={\rho }_{{ab}}^{N}(T)\exp (-b\sqrt{t})$, where ${\rho }_{{ab}}^{F}\left(T\right)$ and ${\rho }_{{ab}}^{N}(T)$ are the fluctuations and normal-state resistivity, respectively; $t=\left(\frac{T}{{T}_{{HN}}}\right)-1$; and b is a fitting parameter on the order of 1. The Halperin-Nelson behavior proves fluctuations of the superconducting order parameter above a 2D Berezinskii–Kosterlitz–Thouless (BKT) transition.

Despite the decrease in *T*_*c*_, the exotic Pauli-breaking Ising superconductivity appeared in monolayer NbSe_2_, few-layer NbSe_2_ and Ba_6_Nb_11_S_28_ vdWs superlattice has attracted intensive attentions^8^. For a superconductor, applying an external magnetic field can suppress its superconductivity either via. the Zeeman effect or through in-plane orbital effects. The Zeeman effect, in particular, imposes an upper bound on the critical magnetic field, known as the Pauli (or Clogston–Chandrasekhar) limit (${\mu }_{0}{H}_{p}=1.84{T}_{c}^{{zero}}$)^42^. The Pauli violation ratio (PVR), defined as *μ*_*0*_*H*_*c‖*_*(0)/μ*_*0*_*H*_*P*_, is generally smaller than 1.0 for conventional superconductors, including bulk NbSe_2_. In monolayer NbSe_2_, Pauli-limit violation Ising-superconductivity can be achieved in the presence of finite-momentum pairing or strong spin–orbit coupling (SOC)^8^. In bulk superlattice Ba_6_Nb_11_S_28_ and few-layer NbSe_2_, some exotic Pauli-breaking Fulde–Ferrell–Larkin–Ovchinnikov (FFLO) state and orbital-FFLO state 2D superconductivity are also reported recently^18,43^. In (NaOH)_0.5_NbSe_2_, we show that its 2D superconducting behavior is more like the Ising-superconductivity appeared in monolayer NbSe_2_. In Fig. S11d, the *μ*_*0*_*H*_*c2*_*-T/T*_*c*_ diagram for (NaOH)_0.5_NbSe_2_ clearly shows that the Pauli violation ratio ${\mu }_{0}{H}_{c2}^{{ab}}$*(0)/*$\,{\mu }_{0}{H}_{p}$ reached a respectable high value up to 4.0, close to that of monolayer NbSe_2_ and well above the PVR values in Ba_6_Nb_11_S_28_ and few-layer NbSe_2_^18,43^. In terms of anisotropy in both the normal state resistivity (β) and superconductivity (γ), the values in (NaOH)_0.5_NbSe_2_ (β_max_ > 2,000,000, γ = 32.5) are much higher than that in the ‘layer-in-cake’ 2D superconductors Ba_6_Nb_11_S_28_ (β_max_ ≈ 2,000, γ = 26.34) and Ba_6_Nb_11_Se_28_ (β_max_ ≈ 20.0, γ = 10.3)^18,31^. Moreover, considering the absence of sharp shapes of ${\mu }_{0}{H}_{c2}^{{ab}}(0)$ data observed in FFLO systems of few-layer NbSe_2_ and Ba_6_Nb_11_S_28_, and the high value of PVR that also exceeds the limit of Rashba-type SOC, it is reasonable to assign the Pauli-breaking superconductivity in (NaOH)_0.5_NbSe_2_ to the effect of Ising paring without FFLO, a mechanism that well acknowledged in monolayer NbSe_2_. Above all, the drastic enhancement of CDW above 110 K as well as the occurrence of exotic Pauli-breaking 2D superconductivity below 1.5 K, consistently suggests that the (NaOH)_0.5_NbSe_2_ bulk crystal behave just like a decoupled NbSe_2_ monolayer.

In conclusion, we have developed a method to fabricate aerogel-like superlattices by incorporating hydrate molecules AOH (A = Na, K) into MX_2_ (M = Ta, Nb; X = S, Se), resulting in large single crystals consisting of ~2 nm thick, porous hydrate layers and MX_2_ monolayers. The elimination of interlayer electron couplings turns the metallic parent materials into ‘insulators’ along the interlayer direction, whereas along other crystallographic axes the metallic state remains. The vanished interlayer electron couplings are also supported by the ideal 2D electronic band structure and fermi surfaces. Fascinatingly, the rarely occupied (5%) hydrate layers also cut off the interlayer vibrational coupling in bulk (NaOH)_0.5_NbSe_2_, leading to an extremely low interlayer coupling energy of 2.5 meV/Å^2^ and interlayer thermal conductivity of 0.28 W m^−1^ K^−1^. The disappearance of the interlayer shearing vibrational model, the appearance of two CDW-related models well above 110 K and the exotic Pauli-breaking 2D superconductivity below 1.5 K are nearly identical to the behaviors found in NbSe_2_ monolayers. Our findings suggest the insertion of highly porous (aerogel-like) block layers can effectively decouple the monolayers in 3D crystal lattices, so that intrinsic 2D properties can be realized in conventional bulk materials.

## Methods

### Sample synthesis

For the first step, MX_2_ (M = Ta, Nb; X = S, Se) crystals were grown from M powder of 99.95 % purity and X pellets of 99.999 % purity by iodine 99.8 % vapor transport in a gradient of 730–700 °C in a sealed quartz tube for 15 days. Then, 0.3–0.6 g of thiourea (alfa, 99.9% purity) and 1–2 g AOH (A = Na, K) (alfa, 99. 9% purity) were dissolved in 10 ml of ammonium sulfide aqueous solution (14 % in water) in a Teflon-lined stainless-steel autoclave (volume 25 mL). Then, several pieces of MX_2_ crystals were added to the solution. Finally, the autoclave was sealed and heated up to 100–130 °C for 130 h. Upon recovery, large (NaOH)_0.5_NbSe_2_ single crystals with a different silver metallic luster were obtained by leaching and clearing.

### Structural characterization and composition determination

Powder X-ray diffraction (PXRD) patterns were collected at room temperature on a Rigaku smart Lab X-ray diffractometer operated at 40 kV voltage and 40 mA current using Cu Ka radiation (λ = 1.5406 Å). The 2θ range was 10–80°with a step size of 0.01. Indexing and Rietveld refinement were performed using the DICVOL91, Fullprof, and MDI Jade programs. Single crystal X-ray diffraction (SCXRD) patterns at 295 K were collected using a Bruker D8 VENTURE PHOTO II diffractometer with multilayer mirror monochromatized Mo Kα (λ = 0.71073 Å) radiation. Unit cell refinement and data merging were performed using the SAINT program, and an absorption correction was applied using multi-Scans scanning. Structural solutions with the P -3 m 1 space groups were obtained by intrinsic phasing methods using the program APEX3, and the final refinement was completed with the Jana 2020 suite of programs. The electron density maps of the two samples were first constructed by the charge flipping method implemented in the Jana2020 software. Scanning electron microscopy (SEM) images were taken on a Phenom pro XL microscope equipped with an electron microprobe analyzer for the semiquantitative elemental analysis in the energy-dispersive X-ray spectroscopy (EDS) mode.

### Magnetic torque measurements

The torque measurements used commercial components provided by Quantum Design Company (TRO Torque Magnetometer). As illustrated in the inset of Fig. S9, the single crystal is mounted onto a piezo-resistive cantilever before measuring. The applied magnetic field is 1 T.

### The density functional theory (DFT) calculations

were performed within the Vienna ab initio simulation package^44^. We adopted the generalized gradient approximation (GGA) in the form of Perdew-Burke-Ernzerhof (PBE) for the exchange-correlation potential^45^. The projector augmented-wave (PAW) pseudopotentials were used with a plane wave energy of 500 eV; 4s24p4 for Se, 4p64d45s1 for Nb, 2p63s1 for Na, 2p42s2 for O and 1s1 for H electron configuration were treated as valence electrons. A Monkhorst-Pack^46^ Brillouin zone sampling grid with a resolution of 0.02 × 2πÅ^−1^ in the self-consistent calculation and 0.01 × 2πÅ^−1^ for Fermi surface calculation were applied. The position of H in (NaOH)_0.5_NbSe_2_ was manually added and the position of H and O were relaxed until all the forces on them were less than 0.1 eV/Å. The lattice constants were derived from the experimental results. The van der Waals interaction was considered using the DFT-D3 method of Grimme^47,48^. The total energy of the system as a function of interlayer separation is calculated by using GGA-PBE (Perdew–Burke–Ernzerhof) functional, as implemented in the CASTEP code. The kinetic energy cutoff for the plane-wave basis set was 410 eV. In the self-consistent potential and total energy calculations of NbSe2 layers, a set of (8 × 8 × 1) k-point samplings was used for Brillouin zone integration. The convergence criterion of self-consistent calculations for ionic relaxations was 10^-4 ^eV between two consecutive steps. By using the conjugate gradient method, all atomic positions and unit cells were optimized until the atomic forces were 0.05 eV/Å.

### Thermal conductivity measurements

The out-of-plane thermal conductivity was measured using time-domain thermoreflectance (34). The pump and probe beam optical beams are focused on the surface of the samples using a microscope objective lens. The thermal conductivity is determined by comparing the time dependence of the ratio of the in-phase V_in_ and the out-of-phase V_out_ signals from the rf lock-in amplifier to calculations using a thermal model. The in-plane thermal conductivity on a (NaOH)_0.5_NbSe_2_ single crystal was measured using a steady-state four-contact method on a custom-built small sample experiment mounted on a furnace which integrates a calibrated heat-pipe in series with the sample.

### Resistivity

Resistivity measurements were carried out using a SQUID PPMS-9 system (2–400 K, 0–9 T) and a home-made low temperature and high magnetic field transport measurement system (300 mK–300 K, 0–18 T). Temperature dependence of the in-plane resistivity ρ_ab_(*T*) of NbSe_2_ and (NaOH)_0.5_NbSe_2_ samples were measured in a standard four-probe configuration with the applied current less than 2 mA. The out-of-plane resistivity ρ_c_(*T*) of NbSe_2_ and (NaOH)_0.5_NbSe_2_ samples were measured using the Montgomery methods. Measurements in magnetic fields up to 18 T were conducted.

### Supplementary information

Supplementary Information

Peer Review File

## Data Availability

The data that support the findings of this study are available from the corresponding authors upon reasonable request.

## References

[1] Novoselov KS (2004). Electric field effect in atomically thin carbon films. Science.

[2] Mak KF, Lee C, Hone J, Shan J, Heinz TF (2010). Atomically thin MoS_2_: a new direct-gap semiconductor. Phys. Rev. Lett..

[3] Splendiani A (2010). Emerging photoluminesence in monolayer MoS_2_. Nano Lett..

[4] Tsen AW (2016). Nature of the quantum metal in a two-dimensional crystalline superconductor. Nat. Phys..

[5] Lei J (2020). High mobility in a van der Waals layered antiferromagnetic metal. Sci. Adv..

[6] Zhao W (2021). Efficient Fizeau drag from Dirac electrons in monolayer graphene. Nature.

[7] Xi X (2015). Strongly enhanced charge-density-wave order in monolayer NbSe_2_. Nat. Nanotechnol..

[8] Xi X (2016). Ising pairing in superconducting NbSe_**2**_ atomic layers. Nat. Phys..

[9] Qiao J (2014). High-mobility transport anisotropy and linear dichroism in few-layer black phosphorus. Nat. Commun..

[10] Huang Y (2020). Universal mechanical exfoliation of large-area 2D crystals. Nat. Commun..

[11] Chen X (2018). CVD-grown monolayer MoS_2_ in bioabsorbable electronics and biosensors. Nat. Commun..

[12] Xiang D, Liu T (2021). Monolayer transistors at wafer scales. Nat. Electron..

[13] Li S (2018). Vapour–liquid–solid growth of monolayer MoS_2_ nanoribbons. Nat. Mater..

[14] Lim B, Rahtu A, Gordon R (2003). Atomic layer deposition of transition metals. Nat. Mater..

[15] Cao Y (2018). Unconventional superconductivity in magic-angle graphene superlattices. Nature.

[16] Cao Y (2018). Correlated insulator behaviors at half-filling in magic-angle graphene superlattices. Nature.

[17] Tongay S (2014). Monolayer behaviors in bulk ReS_2_ due to electronic and vibrational decoupling. Nat. Commun..

[18] Devarakonda A (2020). Clean 2D superconductivity in a bulk van der Waals superlattice. Science.

[19] Ghosh T, Dutta M, Sarkar D, Biswas K (2022). Insights into low thermal conductivity in inorganic materials for thermoelectrics. J. Am. Chem. Soc..

[20] Pierre A, Pajonk G (2002). Chemistry of aerogels and their applications. Chem. Rev..

[21] Bin Y, Lai S, Li J, Li L, Bai S (2021). Trash into treasure: stiff, thermally insulating and highly conductive carbon aerogels from leather wastes for high-performance electromagnetic interference shielding. J. Mater. Chem. C.

[22] Biener J (2011). Advanced carbon aerogels for energy applications. Energy Environ. Sci..

[23] Berthier C, Molinié P, Jérome D (1976). Evidence for a connection between charge density waves and the pressure enhancement of superconductivity in 2H-NbSe_2_. Solid State Commun..

[24] Nakata Y (2018). Anisotropic band splitting in monolayer NbSe_2_: implications for superconductivity and charge density wave. npj 2D Mater. Appl..

[25] Venkata Rami Reddy B, Ravikumar R, Nithy C, Gopukumar S (2015). High performance Na_*x*_CoO_2_ as a cathode material for rechargeable sodium batteries. J. Mater. Chem. A.

[26] Hao Q (2016). A new potassium intercalation compound of 3R-Nb_1.1_S_2_ and its superconducting hydrated derivative synthesized via soft chemistry strategy. ChemistrySelect.

[27] Fan X (2019). Effects of Rb intercalation on NbSe_2_: phase formation, structure, and physical properties. Inorg. Chem..

[28] Naito M, Tanaka S (2018). Electrical transport properties in 2*H*-NbS_2_, -NbSe_2_, -TaS_2_ and -TaSe_2_. J. Phys. Soc. Jpn..

[29] Klemm R (2016). Pristine and intercalated transition metal dichalcogenide superconductors. Physica C.

[30] Meyer SF, Howard RE, Stewart GR, Acrivos JV, Geballe TH (1975). Properties of intercalated 2H-NbSe_2_, 4H-TaS_2_, and 1T-TaS_2_. J. Chem. Phys..

[31] Ma K (2022). two-dimensional superconductivity in a bulk superlattice van der Waals material Ba_6_Nb_11_Se_28_. Phys. Rev. Mater..

[32] Calandra M, Mazin I, Mauri F (2009). Effect of dimensionality of the charge-density wave in few-layer 2H-NbSe_2_. Phys. Rev. B.

[33] Jiang P, Qian X, Yang R (2018). Tutorial: time-domain thermoreflectance (TDTR) for thermal property characterization of bulk and thin film materials. J. Appl. Phys..

[34] Ren Q (2023). Extreme phonon anharmonicity underpins superionic diffusion and ultralow thermal conductivity in argyrodite Ag_8_SnSe_6_. Nat. Mater..

[35] Jang, H., Ryder, C., Wood, J., Hersam, M. & Cahill, D., 3D anisotropic thermal conductivity of exfoliated rhenium disulfide. *Adv. Mater*. **29**, 1700650 (2017). 10.1002/adma.201700650 28722239

[36] Acharyya P (2022). Glassy thermal conductivity in Cs3Bi2I6Cl3 single crystal. Nat. Commun..

[37] Mukhopadhyay S (2018). Two-channel model for ultralow thermal conductivity of crystalline Tl_3_VSe_4_. Science.

[38] Kim SE (2021). Extremely anisotropic van der Waals thermal conductors. Nature.

[39] Ho CY, Powell RW, Liley PE (1972). Thermal conductivity of the elements. J. Phys. Chem. Ref. Data.

[40] Ding H (2016). Computational approach for epitaxial polymorph stabilization through substrate selection. ACS Appl. Mater. Interfaces.

[41] Halprin B, Nelson D (1978). Theory of two-dimensional melting. Phys. Rev. Lett..

[42] Clogston A (1962). Upper limit for the critical field in hard superconductors. Phys. Rev. Lett..

[43] Wan P (2023). Orbital Fulde–Ferrell–Larkin–Ovchinnikov state in an Ising superconductor. Nature.

[44] Kresse G, Furthmuller J (1996). Efficiency of ab-initio total energy calculations for metals and semiconductors using a plane-wave basis set. Comput. Mater. Sci..

[45] Perdew JP, Burke K, Ernzerhof M (1996). Generalized gradient approximation made simple. Phys. Rev. Lett..

[46] Monkhorst HJ, Pack JD (1976). Special points for Brillouin-zone integrations. Phys. Rev. B.

[47] Grimme S, Ehrlich S, Goerigk L (2011). Effect of the damping function in dispersion corrected density functional theory. J. Comput. Chem..

[48] Grimme S, Antony J, Ehrlich S, Krieg H (2011). A consistent and accurate ab initio parametrization of density functional dispersion correction (DFT-D) for the 94 elements H-Pu. J. Chem. Phys..

[49] Zhou J (2019). 2DMatPedia, an open computational database of two-dimensional materials from top-down and bottom-up approaches”. Sci. Data.

[50] Jang H, Wood J, Ryder C, Hersam M, Cahill D (2015). Anisotropic thermal conductivity of exfoliated black phosphorus. Adv. Mater..

[51] Song, N. et al. Highly Anisotropic Thermal Conductivity of Layer-by-Layer Assembled Nanofibrillated Cellulose/Graphene Nanosheets Hybrid Films for Thermal Management. *ACS Appl. Mater. Interfaces* **9**, 2924–2932 (2017). 10.1021/acsami.6b11979 28045485

