# Supplementary material for: High anisotropy in electrical and thermal conductivity through the design of aerogel-like superlattice (NaOH)0.5NbSe2
Source: Nat Commun. 2023 Oct 21;14:6689. doi: 10.1038/s41467-023-42510-0 (PMC10590432; doi:10.1038/s41467-023-42510-0)
Supplement: Supplementary file 1 — Supplementary Information [file 41467_2023_42510_MOESM1_ESM.pdf]

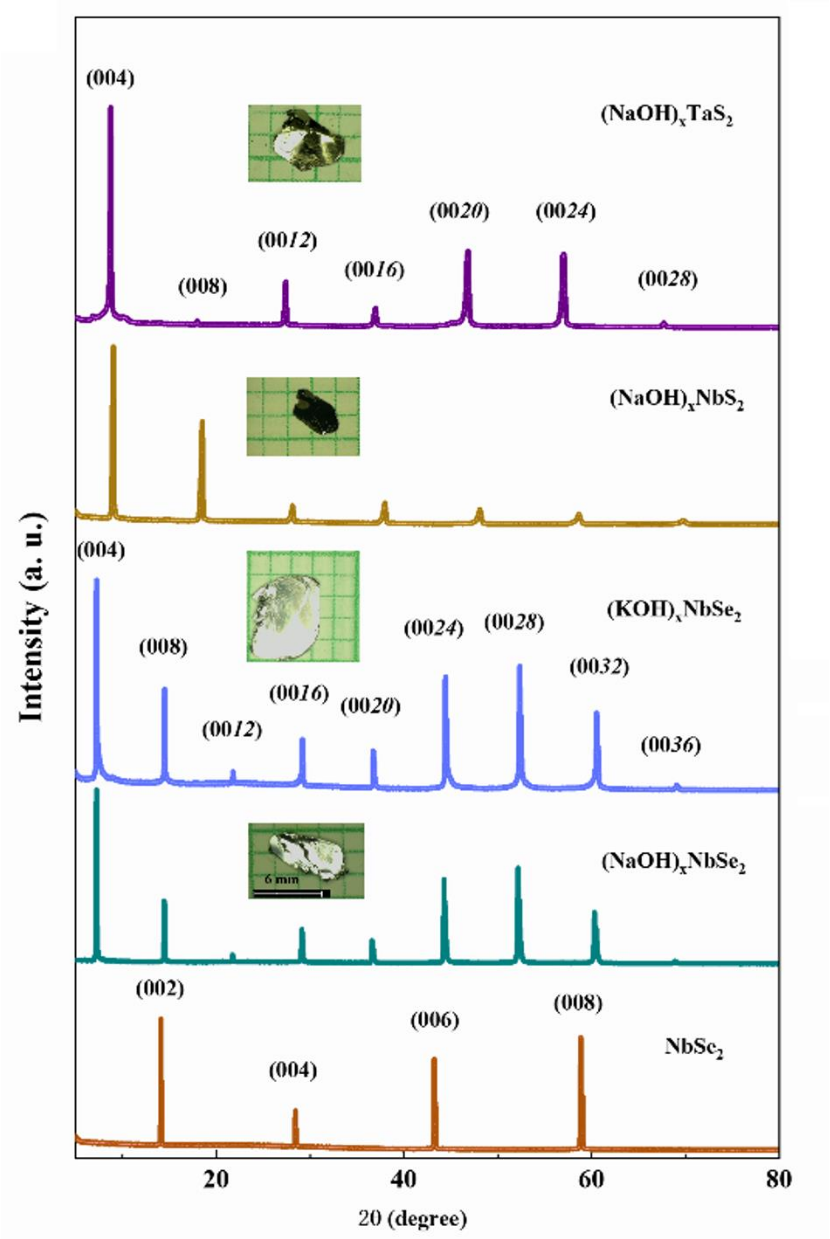

Figure S1. Powder X-ray diffraction patterns along (00l) direction for $NbSe_2$ and a series of $(AOH)_xMX_2$ (Na, K; M=Ta, Nb; X=S, Se).

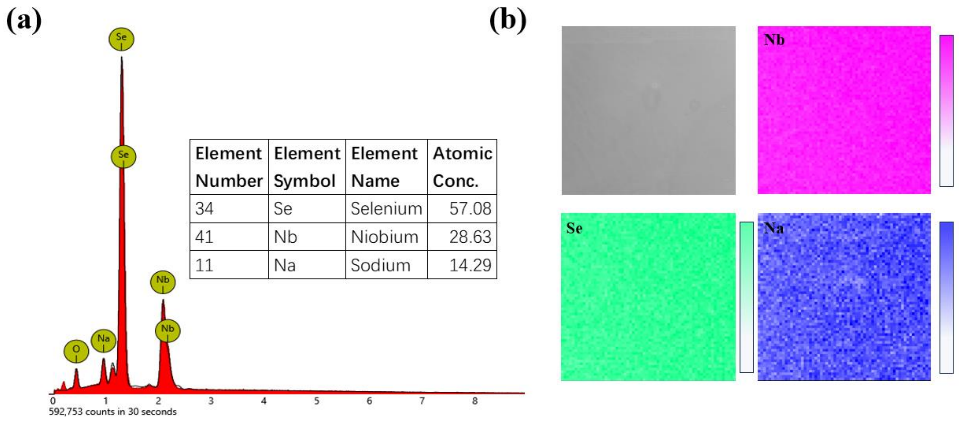

Element Number	Element Symbol	Element Name	Atomic Conc.
34	Se	Selenium	57.08
41	Nb	Niobium	28.63
11	Na	Sodium	14.29

Figure S2. EDS spectra and chemical composition analysis for $(NaOH)_{0.5}NbSe_2$. (a) EDS spectra for $(NaOH)_{0.5}NbSe_2$. (b) Chemical composition analysis for $(NaOH)_{0.5}NbSe_2$.

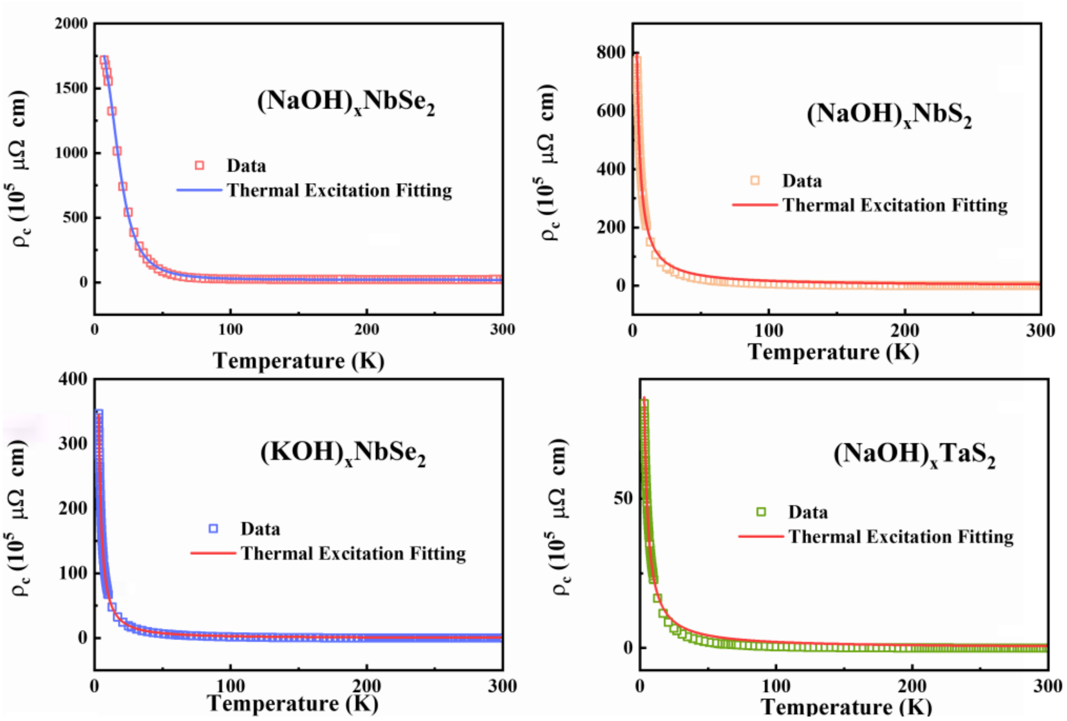

Figure S3. The 3-300 K out-of-plane resistivity-temperature curve for $(AOH)_xMX_2$ (A=Na, K; M=Nb, Ta, X=S, Se).

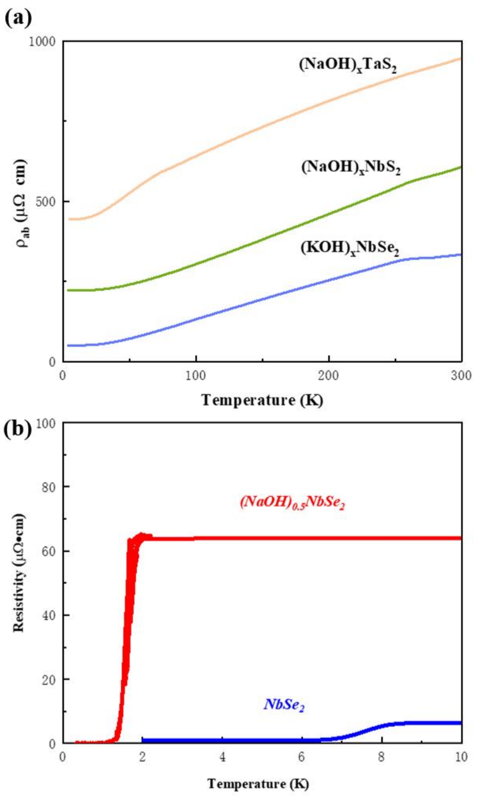

Figure S4. Transport properties for $(AOH)_xNbSe_2$. (a) The 3-300 K in-plane resistivity for $(KOH)_xNbSe_2$, $(NaOH)_xNbS_2$, $(NaOH)_xTaS_2$. (b) The in-plane resistivity for $(NaOH)_{0.5}NbSe_2$ and $NbSe_2$ below 10 K, where the superconducting transition is clearly observed.

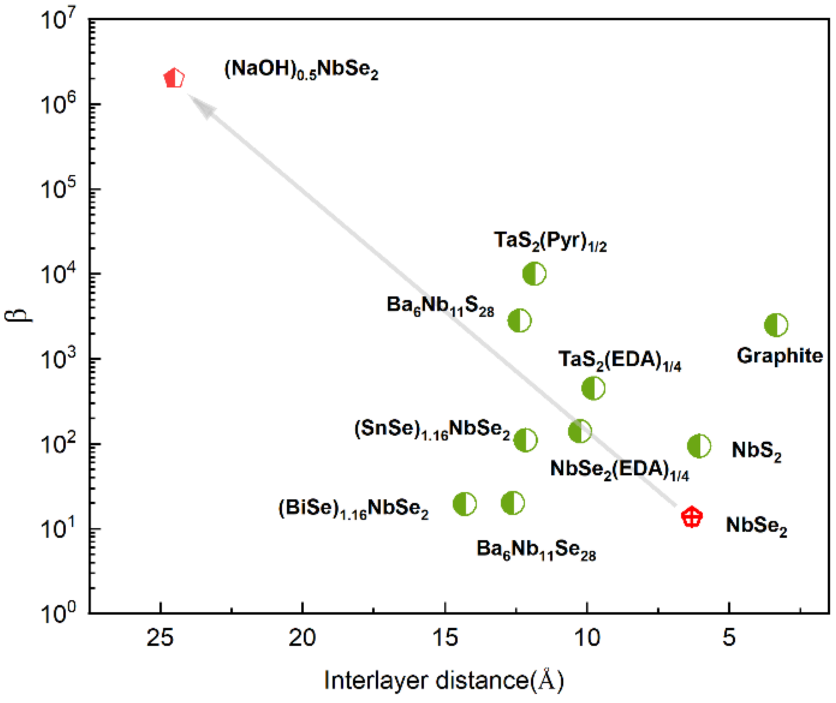

Figure S5. The interlayer distance and resistivity anisotropy β in graphite, $(NaOH)_{0.5}NbSe_2$, different metallic TMD materials and their intercalated phases.

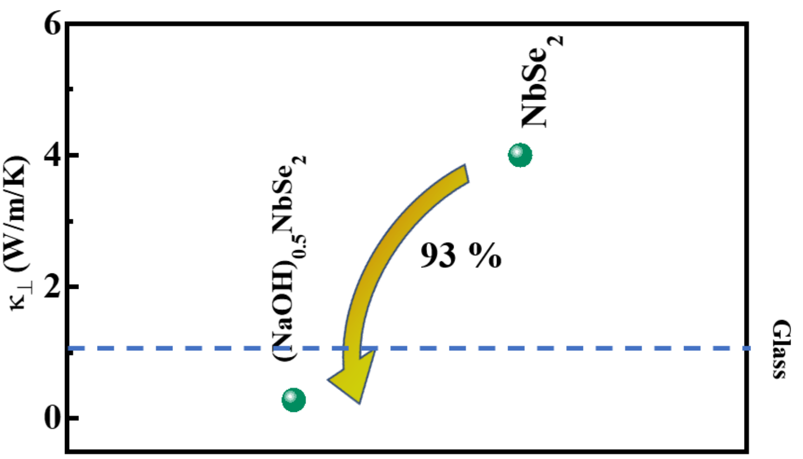

Figure S6. The out-of-plane thermal conductivity of $(NaOH)_{0.5}NbSe_2$ and bulk $NbSe_2$.

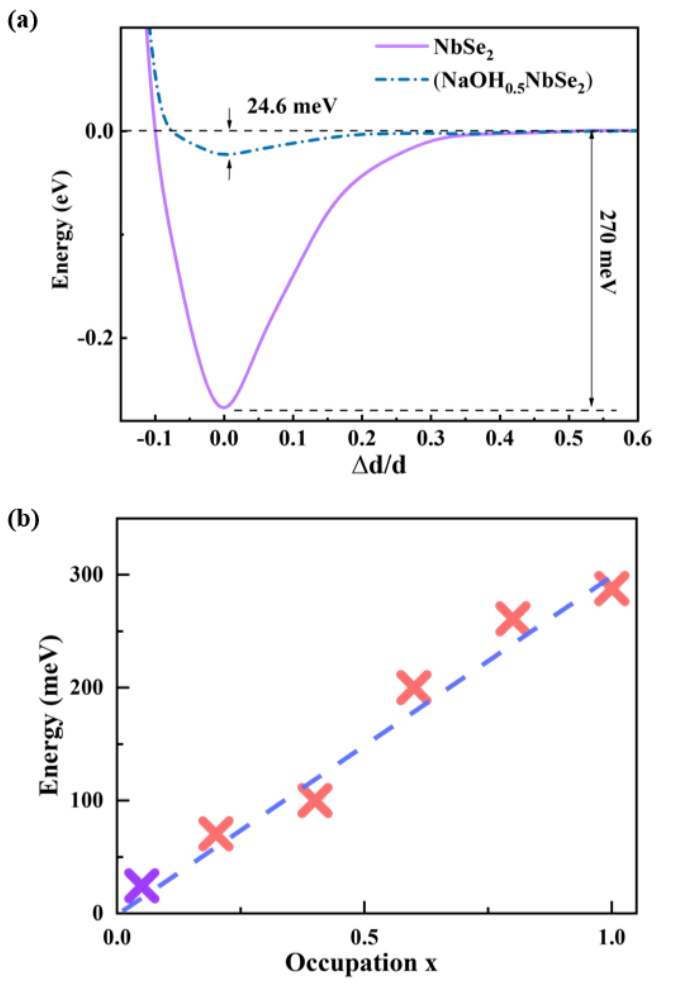

Figure S7. Calculated results for $(NaOH)_{0.5}NbSe_2$ and bulk $NbSe_2$**.** (a) The calculated interlayer coupling energy of $(NaOH)_{0.5}NbSe_2$ and bulk $NbSe_2$. (b) The calculated interlayer coupling energy of the system as a function of NaOH occupation factor x.

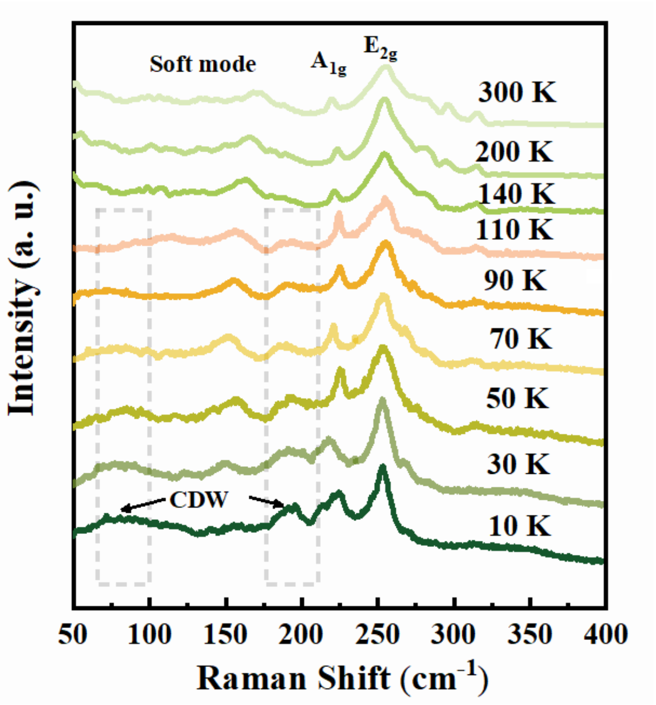

Figure S8. The temperature-dependent Raman spectra for $(NaOH)_{0.5}NbSe_2$.

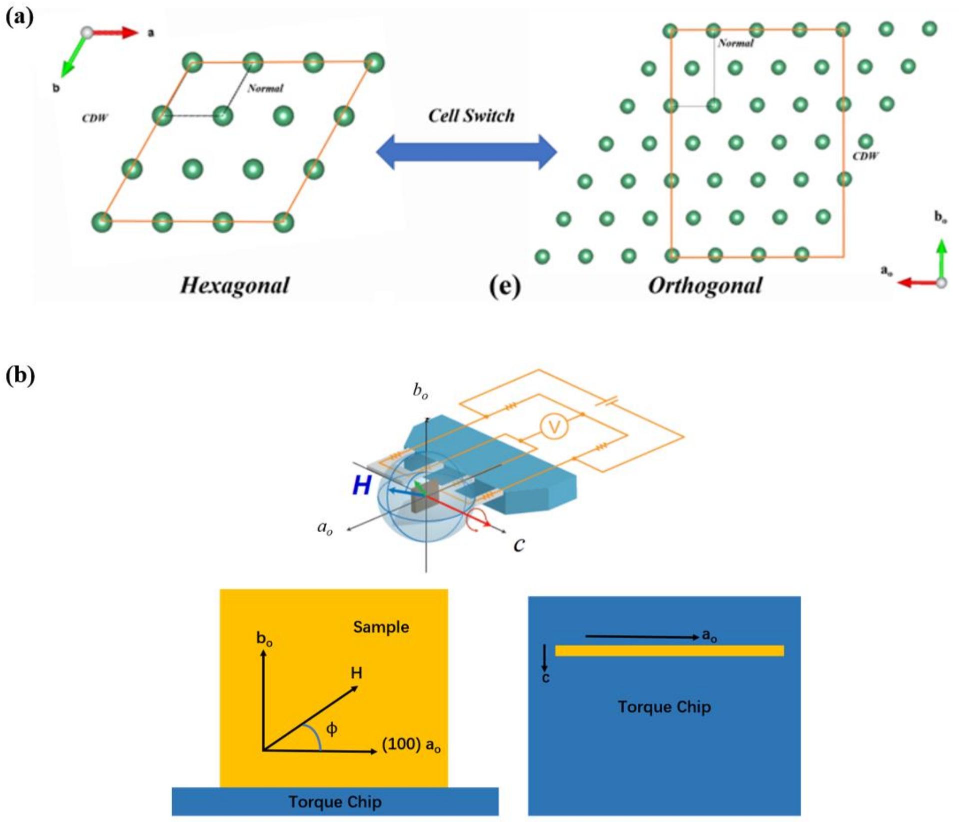

Figure S9. Scheme for Torque measurements. (a) Scheme of the symmetry switch from hexagonal system to the orthorhombic for the CDW order in the Nb atomic layer. (b) Schematic diagram of the Torque measurements.

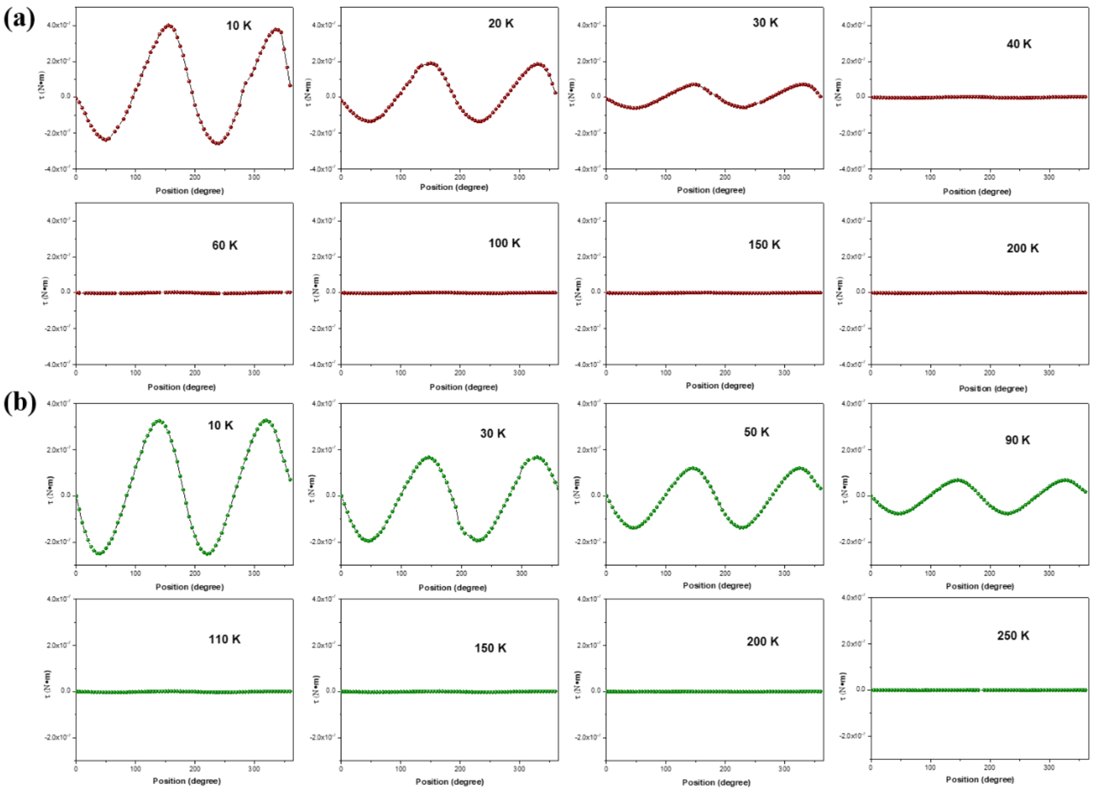

Figure S10. Raw data for torque measurements. (a) Raw data for the torque measurements of (a) $NbSe_2$ and (b) $(NaOH)_{0.5}NbSe_2$ under different temperatures.

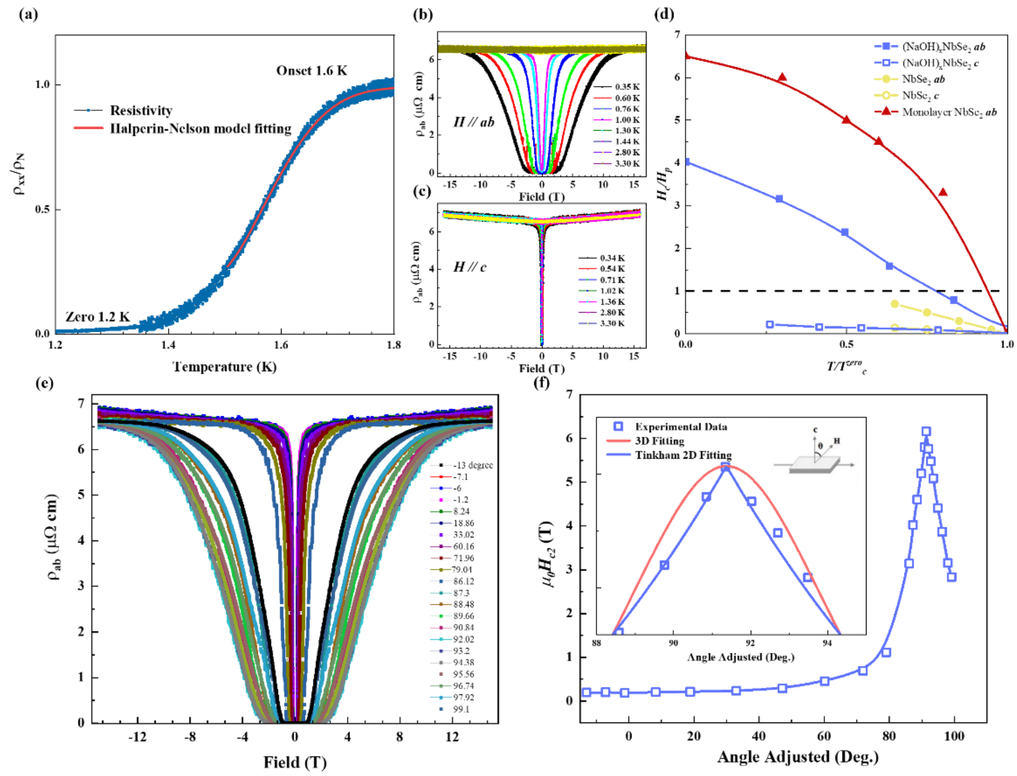

Figure S11. Superconducting properties for $(NaOH)_{0.5}NbSe_2$ (a) Resistivity as a function of temperature ρ_{ab} (T) for $(NaOH)_{0.5}NbSe_2$, ρ_{ab} (T) is fit by the Halperin-Nelson model in red line. (b&c) Field dependence of ρ_{ab} (H) was measured at various temperatures in dc magnetic fields up to 18 T for (b) H||ab and (c) H||c, the applied current is 100 μA. (d) The critical field H_{c2}/H_P as a function of transition temperature $T_C/\ T_c^{zero}$ for $(NaOH)_{0.5}NbSe_2$, bulk $NbSe_2$, and $NbSe_2$ monolayer. The dashed line corresponds to the Pauli paramagnetic limit. (e) Field dependence of ρ_{ab} (H) was measured at various angles in dc magnetic fields up to 15 T at 0.4 K, the applied current is 100 μA. (f) Angular dependence of upper critical field μ_0H_{c2} measured with fits to the 2D-Tinkham model. In the inset, the solid points are experimental data, whereas the red and black curves are fitting to 2D Tinkham and G-L model, θ is the angle between the magnetic field and the sample normal direction. We define μ_0H_{c2} and its error to be when ρ_{ab} reaches 50 ± 5% of the normal-state value.

Table S1 Crystallographic data of $(NaOH)_{0.5}NbSe_2$(refinement of SCXRD Data)

**Formula**			**Na Nb1.98 O Se4**			
**Formula Weight**			**535.50**			
**Space group**			***P -3 m 1***			
**a, c(Å)**			**3.4554(1), 48.9648(2)**			
**Z**			**1**			
**Crystal size(μm^3)**			**132 * 74 * 17**			
**Temperature(K)**			**298**			
**Radiation(Å)**			**Mo-Kα λ = 0.71073**			
**R_1, wR_2, S**			**0.0313, 0.0615, 1.0946**			
**Atomic positions and equivalent displacement parameters**						
**Atom**	**Muti.**	**x**	**y**	**z**	**Occ.**	**U_{iso}**
**Se1**	**2**	**0.667**	**0.333**	**0.2151(1)**	**1**	**0.019(11)**
**Se2**	**2**	**0.667**	**0.333**	**0.2854(13)**	**1**	**0.020(2)**
**Nb**	**2**	**1**	**0**	**0.2476(2)**	**0.989(12)**	**0.016(12)**
**Na1**	**1**	**0**	**0**	**0**	**0.404(2)**	**0.045(11)**
**O1**	**2**	**0.667**	**0.333**	**0.0362(11)**	**0.197(14)**	**0.015(2)**
**Na2**	**2**	**0**	**0**	**0.1247(13)**	**0.051(2)**	**0.012(11)**
**O2**	**2**	**0.667**	**0.333**	**0.1613(19)**	**0.049(11)**	**0.041(14)**
**Na3**	**1**	**0**	**0**	**0.5**	**0.406(1)**	**0.043(4)**
**O3**	**2**	**0.667**	**0.333**	**0.5367(12)**	**0.208(13)**	**0.021(2)**
**Na4**	**2**	**0**	**0**	**0.3752(11)**	**0.049(12)**	**0.017(14)**
**O4**	**2**	**0.667**	**0.333**	**0.3393(14)**	**0.051(2)**	**0.036(2)**

Table S2 Selected bond length and bond angle for $(NaOH)_{0.5}NbSe_2$, 2H-$NbSe_2$ (PDF card-04-001-9255) and NaOH (PDF card-00-045-0744).

	$(NaOH)_{0.5}NbSe_2$	2H-$NbSe_2$	NaOH
**Bond length (Å)**			
**Nb-Se**	**2.552(7)**	**2.53579(1)**	**----**
**Na-O**	**2.66(6)**	**----**	**2.6391(6)**
**Bond angle (deg)**			
**Se-Nb-Se**	**85.2(3)**	**85.63 (08)**	**----**
**O-Na-O**	**80.3(15)**	**----**	**83.71(2)**

Table S3 The Debye-Einstein Model various fitting parameters:

$$Cp = 9aNR\left(\frac{T}{\theta_{Debye}}\right)^3 Debye(T) + \frac{3(1-a)bNR\left(\frac{\theta_{E1}}{T}\right)^2 e^{\left(\frac{\theta_{E1}}{T}\right)}}{e^{2\left(\frac{\theta_{E1}}{T}-1\right)}} + \frac{3(1-a)(1-b)NR\left(\frac{\theta_{E2}}{T}\right)^2 e^{\left(\frac{\theta_{E2}}{T}\right)}}{e^{2\left(\frac{\theta_{E2}}{T}-1\right)}}$$

Where *N*, θ_{Debye} and $\theta_{E1(E2)}$ are Avogadro number, characteristic Debye temperature and characteristic Einstein temperature respectively. The parameter *a* is the proportion of Debye model.

θ_{Debye}	θ_{E1}	θ_{E2}	*a*	*b*
**237 K**	**128 K**	**174 K**	**0.09**	**0.31**
